# RETRACTION: Effect of Enhanced Recovery after Surgery Nursing on the Incidence of Surgical Site Wound Infection in Older Adults With Hip Fractures: A Meta‐Analysis

**DOI:** 10.1111/iwj.70268

**Published:** 2025-03-03

## Abstract

**RETRACTION:** LuX. and LiJ. , “Effect of Enhanced Recovery after Surgery Nursing on the Incidence of Surgical Site Wound Infection in Older Adults With Hip Fractures: A Meta‐Analysis,” International Wound Journal 21, no. 2 (2024): e14436, 10.1111/iwj.14436.PMC1082871637885328

The above article, published online on 26 October 2023, in Wiley Online Library (http://onlinelibrary.wiley.com/), has been retracted by agreement between the journal Editor in Chief, Professor Keith Harding; and John Wiley & Sons Ltd. Following an investigation by the publisher, all parties have concluded that this article was accepted solely on the basis of a compromised peer review process. The editors have therefore decided to retract the article. The authors did not respond to our notice regarding the retraction.

**RETRACTION:** X. Lu and J. Li , “Effect of Enhanced Recovery after Surgery Nursing on the Incidence of Surgical Site Wound Infection in Older Adults With Hip Fractures: A Meta‐Analysis,” International Wound Journal 21, no. 2 (2024): e14436, 10.1111/iwj.14436.PMC1082871637885328

The above article, published online on 26 October 2023, in Wiley Online Library (http://onlinelibrary.wiley.com/), has been retracted by agreement between the journal Editor in Chief, Professor Keith Harding; and John Wiley & Sons Ltd. Following an investigation by the publisher, all parties have concluded that this article was accepted solely on the basis of a compromised peer review process. The editors have therefore decided to retract the article. The authors did not respond to our notice regarding the retraction.

